# Sevoflurane preconditioning in living liver donation is associated with better initial graft function after pediatric transplantation: a retrospective study

**DOI:** 10.1186/s13741-024-00367-x

**Published:** 2024-02-28

**Authors:** Audrey Dieu, Loïc Benoit, Candice Dupont, Catherine de Magnée, Raymond Reding, Thierry Pirotte, Arnaud Steyaert

**Affiliations:** 1 Department of Anesthesiology, Cliniques Universitaires Saint Luc, Université Catholique de Louvain, Avenue Hippocrate 10, Brussels, 1200 Belgium; 2 Department of General and Pediatric Surgery, Cliniques Universitaires Saint-Luc, Brussels, Belgium

**Keywords:** Sevoflurane, Ischemia-reperfusion injury, Living donor liver transplantation, Initial poor graft function, Anesthesia

## Abstract

**Introduction:**

Initial allograft function determines the patient’s immediate prognosis in pediatric liver transplantation. Ischemia-reperfusion injuries play a role in initial poor graft function (IPGF). In animal studies, preconditioning with inhaled anesthetic agents has demonstrated a protective effect on the liver. In humans, the few available studies are conflicting. This study assesses the association between the hypnotic agent used to maintain anesthesia during hepatectomy in living donors and the occurrence of IPGF after pediatric transplantation.

**Methods:**

We conducted a single-center retrospective analysis of children who received a living donor liver transplant (LDLT) between 2010 and 2019. We analyzed the incidence of EAD according to the hypnotic agent used to maintain general anesthesia during donor hepatectomy.

**Results:**

We included 183 pairs of patients (living donors-recipients). The anesthetics used in the donor were propofol (*n* = 85), sevoflurane (*n* = 69), or propofol with sevoflurane started 30 min before clamping (*n* = 29). Forty-two children (23%) developed IPGF. After multivariate logistic regression analysis, factors significantly associated with the occurrence of IPGF were the anesthesia maintenance agent used in the donor (*p* = 0.004), age of the donor (*p* = 0.03), duration of transplant surgery (*p* = 0.009), preoperative receiver neutrophil to lymphocyte ratio (*p* = 0.02), and albumin (*p* = 0.05).

**Conclusion:**

Significantly fewer children who received a graft from a donor in whom only sevoflurane was used to maintain anesthesia developed IPGF. Although additional research is needed, this preconditioning strategy may provide an option to prevent IPGF after living liver donation.

**Supplementary Information:**

The online version contains supplementary material available at 10.1186/s13741-024-00367-x.

## Introduction

Liver transplantation is the only curative option for end-stage liver disease. Over the last few decades, the improvement of surgical techniques, the refinement of immunosuppressive regimens, and, more generally, the progress made in perioperative care have reduced morbidity and mortality in transplanted patients (Clavien et al. 2007).

Nevertheless, both in adult and pediatric liver transplantation, the risk factors associated with postoperative complications, such as initial poor graft function (IPGF), are not yet clearly identified. The degree and severity of ischemia-reperfusion injury (IRI) significantly impact the early recovery of graft function, which determines the patient’s immediate prognosis (Briceno and Ciria 2010; Lee et al. 2016; Olthoff et al. 2010).

Several protective strategies have been developed to limit the harmful effects of IRI. In several animal studies, most of which were carried out in rats, sevoflurane administration had a protective effect against IRI (Figueira et al. 2019; Granja et al. 2016; Xu et al. 2016). While its mechanisms of action remain only partially understood, proposed explanations include an anti-inflammatory effect and an action on the mitochondrial respiratory chain. Nitric oxide also seems to play a part in this phenomenon (Beck-Schimmer et al. 2008; Datta et al. 2014; Zhou et al. 2013).

A protective intervention can be initiated before the beginning of the ischemia phase (preconditioning) or immediately after reperfusion (postconditioning). Therefore, in transplantation, preconditioning occurs in the donor and postconditioning in the recipient. Studies investigating such interventions are scarce in humans, both in the fields of liver resection and liver transplantation, and results are conflicting (Beck-Schimmer et al. 2015; Benoit et al. 2023; Gajate Martin et al. 2016; Minou et al. 2012). So far, only one study has evaluated the effect of pharmacological preconditioning on the incidence of early allograft dysfunction (EAD) in the context of adult liver transplantation by administering sevoflurane to brain-dead donors (Minou et al. 2012). To our knowledge, no study has ever investigated sevoflurane preconditioning in living donation.

This single-center retrospective cohort study aims to evaluate the association between the hypnotic agent used to maintain anesthesia during hepatectomy in the adult living donor and the occurrence of IPGF in the recipient child.

## Methods

### Patient selection

We reviewed our departmental database to identify pairs of patients (living donors and child recipients) who had undergone the coupled procedures of hepatectomy and transplantation between January 2010 (database inception) and December 2019 (data collection).

Exclusion criteria included a prior history of liver transplantation in the child, ABO-incompatible transplant, small-for-size graft (defined as graft-to-recipient weight ratio < 1%), and patients who had notified their refusal to share data for biomedical research purposes. We also excluded children who developed a thrombotic event of the hepatic artery or the portal vein, as these conditions complicated the adequate interpretation of a rise in transaminase levels.

### Procedures

Routine monitoring used in donors and recipients included arterial oxygen saturation, electrocardiogram, and invasive blood pressure. In the donors, we also monitored the depth of anesthesia through processed electroencephalography and cerebral oxygenation through near-infrared spectroscopy. Depending on the anesthesiologist’s preference, anesthesia in the donor was maintained with either propofol, propofol with the addition of sevoflurane (0.5 to 1 MAC) for 20 to 30 min before the ischemia phase, or sevoflurane alone. The use of adjuvants (sufentanil, clonidine, ketamine) was left to the choice of the senior anesthesiologist in charge of the patient. In both donors and recipients, personalized fluid management was based on a goal-directed strategy. The donors received rocuronium or atracurium for muscle relaxation, while all children received atracurium. Epidural analgesia was provided to all donors unless they refused. Anesthesia was maintained with sevoflurane in all recipients.

### Data collection

The data collected from the donors were age, hypnotic agent used to maintain general anesthesia, adjuvant analgesics administered, and noradrenalin requirements during the surgical procedure.

The data collected from the recipient were age, weight, height, etiology, and severity of the end-stage liver failure using PELD-score (Pediatric End-stage Liver Disease), whether or not there was portal hypertension (PHT), biochemical data (ALAT, ASAT, bilirubin, INR, blood urea nitrogen, creatinine, albumin, and neutrophil-lymphocyte ratio) from the day before surgery to postoperative day 7, peak intraoperative lactate level, graft weight, durations of ischemia phase and surgery, intraoperative vasopressor need and doses, and occurrence of a reperfusion syndrome, defined as a decrease of at least 30% in mean arterial pressure for at least 1 min during the 5 min following reperfusion.

### Statistical analysis

We first summarized the data for the variables relevant to this analysis and analyzed missing values in the dataset according to recently published recommendations (Bartlett et al. 2015; Lee et al. 2021). We then used descriptive statistics to present the included cohort, whose characteristics we report as median [IQR] or numbers (%). We tested the data for normality with the Shapiro-Wilk test. We used chi-square (categorical variables) or Wilcoxon (continuous variables) tests to compare baseline and intraoperative characteristics according to the donor’s anesthetic maintenance agent.

Our primary outcome was the occurrence of IPGF, defined in our pediatric population as the presence of at least one of the following criteria: ASAT or ALAT peak ≥ 1000 U/L during the first week, total bilirubin ≥ 10 mg/dL on postoperative day 7, INR ≥ 1.6 on postoperative day 7. We used univariate logistic regression to assess the relationship between the studied factors and IPGF and estimate odds ratios (ORs) with 95% confidence intervals. To identify factors independently associated with IPGF, we performed multivariate logistic regression after variable selection using a backward stepwise method (entry criteria: *p* < 0.2 in the unadjusted model, exit criteria: *p* > 0.1). For all analyses, we considered a *p* value of 0.05 to be statistically significant.

We performed all statistical analyses with JMP Pro version 16.0 (SAS Institute Inc., Cary, NC, USA).

## Results

### Cohort description

**Fig. 1:**
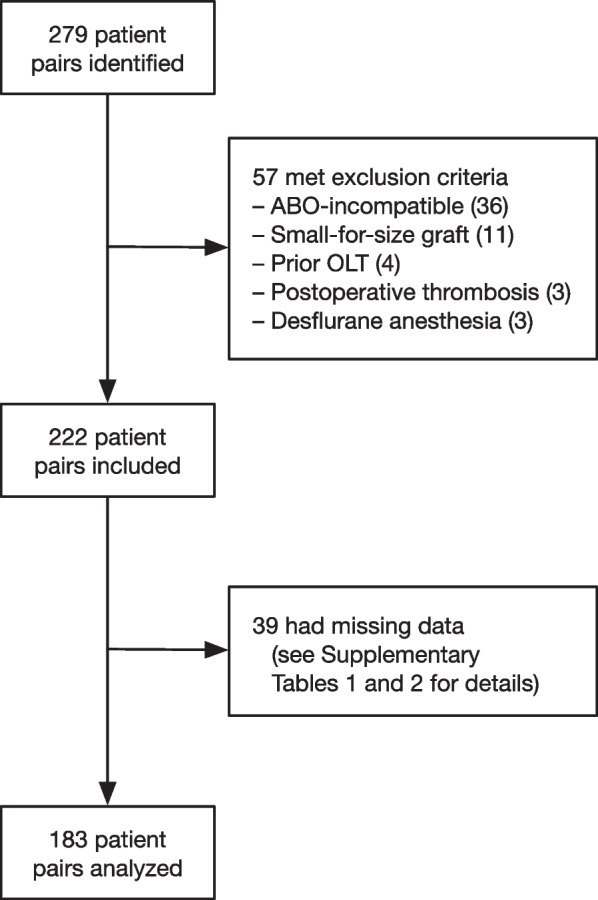
Study flowchart

Figure 1 details the study flowchart. We identified 279 consecutive pairs of patients, of which 57 met one of the exclusion criteria. Of the 222 included pairs, 183 (82.2%) had no missing data for all variables of interest and were identified as complete cases (Supplementary Table S1). Logistic regression showed that noradrenalin use in the donor was associated with the probability of being a complete case (Supplementary Table S2). However, neither the outcome (IPGF), the donor anesthetic agent, nor any other potentially confounding variables were associated with the probability of being a complete case. In this situation, a complete case analysis should provide unbiased estimates (Bartlett et al. 2015). Therefore, we analyzed the 183 patient couples (living donors and recipients) with complete data (Fig. 1).

Forty-two children (23%) developed IPGF. Sixty-nine donors (37.7%) benefited from the maintenance of anesthesia with sevoflurane alone. Eighty-five donors (46.4%) received a continuous infusion of propofol alone. A mixed strategy consisting of the adjunction of 0.5 to 1 MAC of sevoflurane to a continuous infusion of propofol 20 to 30 min before the beginning of the ischemia phase was applied in 29 donors (15.8%).

The preoperative characteristics were similar in the three groups and are reported in Table 1. The intraoperative variables are presented in Table 2. Donors in the sevoflurane and propofol-sevoflurane groups were significantly more likely to have received NSAIDs, sufentanil, and noradrenaline, while the reverse was true for clonidine. The recipients’ ischemia time was significantly longer in the propofol than in the sevoflurane group, while the graft-to-recipient weight ratio was significantly smaller in the propofol-sevoflurane than in the other two groups.

**Table 1:** Preoperative characteristics of the study cohort, according to the anesthetic agent used in the donor

	**Sevoflurane (*n* = 69)**	**Propofol and sevoflurane (*n* = 29)**	**Propofol (*n* = 85)**	***P* value**
**Donor**				
Age (years)	34.3 [29.4–39.0]	33.0 [30.2–36.8]	33.8 [27.7–36.4]	.19
Sex				.94
Female	31 (44.9)	14 (48.3)	40 (47.1)	
Male	38 (55.1)	15 (51.7)	45 (52.9)	
**Recipient**				
Age (years)	1.3 [0.8–2.4]	2.3 [0.9–4.3]	1.5 [0.9–2.7]	.13
Sex				.94
Female	40 (58.0)	16 (55.2)	47 (55.3)	
Male	29 (42.0)	13 (44.8)	38 (44.7)	
Weight (kg)	9.4 [7.7–12.4]	12.0 [8.7–15.9]	9.1 [7.4–14.0]	.15
Liver pathology				.10
Biliary atresia	51 (73.9)	16 (55.2)	51 (60.0)	
Other	18 (26.1)	13 (44.8)	18 (40.0)	
Cirrhosis	59 (85.5)	26 (89.7)	72 (84.7)	.80
ASAT (U/L)	167 [92–226]	171 [87–249]	144 [82–229]	.97
ALAT (U/L)	82 [47–124]	119 [38–148]	87 [49–128]	.61
Bilirubin (mg/dL)	16.1 [2.7–21.5]	13.5 [2.9–22.4]	14.2 [2.9–19.7]	.76
INR	1.28 [1.11–1.67]	1.19 [1.03–1.57]	1.31 [1.08–1.58]	.38
Urea (mg/dL)	15 [11–19]	17 [13–26]	17 [14–22]	.14
Creatinin (mg/dL)	0.24 [0.19–0.31]	0.32 [0.19–0.39]	0.24 [0.19–0.34]	.16
Albumin (g/L)	33 [29–38]	34 [31–41]	32 [28–37]	.27
NLR	0.9 [0.7–1.6]	1.1 [0.6–1.4]	1.1 [0.6–1.7]	.51

Data are presented as median [IQR] or numbers (%). Comparisons between groups (sevoflurane, propofol + sevoflurane, and propofol) with a Kruskal-Wallis test (continuous data) or a chi-square (categorical data)

*Abbreviations*: *ASAT* Aspartate aminotransferase, *ALAT* Alanine aminotransferase, *NLR* Neutrophil/lymphocyte ratio

**Table 2:** Intraoperative characteristics of the study cohort, according to the anesthetic agent used in the donor

	**Sevoflurane (*n* = 69)**	**Propofol and sevoflurane (*n* = 29)**	**Propofol (*n* = 85)**	***P* value**
**Donor**				
NSAIDs	43 (62.3)	27 (93.1)	73 (85.9)	**.0002**
Sufentanil	63 (91.3)	0 (0)	8 (9.4)	**< .0001**
Clonidine	28 (40.6)	27 (93.1)	81 (92.3)	**< .0001**
Ketamine	65 (94.2)	29 (100)	84 (98.3)	.06
Epidural	57 (82.6)	23 (79.3)	72 (84.7)	.79
Noradrenalin	39 (56.5)	6 (20.7)	6 (5.9)	**< .0001**
**Recipient**				
Procedure duration (min)	661 [607–722]	664 [598–702]	647 [585–691]	.37
Ischemia duration (min)	130 [109–155]	143 [124–177]	155 [131–179]	**.003**
Vasopressor use	62 (89.9)	29 (100)	67 (78.9)	**.002**
Lactate peak (mmol/L)	5.2 [3.9–7.0]	5.1 [4.4–6.3]	5.1 [4.1–7.1]	.83
Reperfusion syndrome	13 (18.8)	3 (10.3)	15 (17.6)	.54
Graft weight/body weight (%)	2.9 [2.0–3.7]	2.2 [1.6–3.0]	2.7 [2.0–3.6]	**.02**

Data are presented as median [IQR] or numbers (%). Comparisons between groups (sevoflurane, propofol + sevoflurane, and propofol) with a Kruskal-Wallis test (continuous data) or a chi-square (categorical data)

*Abbreviation*: *NSAIDs* Non-steroidal anti-inflammatory drugs

### Factors associated with initial poor graft function in univariate analysis

The preoperative characteristics of donors and recipients, according to the occurrence of IPGF, are presented in Table 3. Four variables were significantly associated with the risk of developing IPGF: the donor’s age, creatinine levels, NLR as well as the age of the recipient. Additionally, we identified three intraoperative factors significantly associated with IPGF occurrence: the hypnotic agents used to maintain anesthesia in the donor, the duration of the surgery in the recipient, and the graft-to-recipient weight ratio (Table 4).

**Table 3:** Preoperative characteristics of the whole cohort and unadjusted odds ratios for initial poor graft function

	**Whole cohort (*n* = 183)**	**No IPGF (*n* = 141)**	**IPGF (*n* = 42)**	**OR for IPGF (95%CI)**	***P* value**
**Donor**					
Age (years)	34.0 [28.8–37.1]	32.7 [28.1–36.8]	34.7 [31.1–38.5]	1.06 (1.01–1.12)	**.03**
Sex (female)	85 (46.4)	65 (46.1)	20 (47.6)	1.06 (0.53–2.12)	.86
**Receiver**					
Age (years)	1.6 [0.9–2.8]	1.3 [0.8–2.3]	2.3 [1.0–4.9]	1.13 (1.01–1.26)	**.04**
Sex (female)	103 (56.3)	81 (57.4)	22 (52.4)	1.22 (0.62–2.45)	.56
Weight (kg)	9.6 [7.6–14.0]	9.2 [7.4–12.8]	12.7 [7.8–17.8]	1.04 (0.99–1.09)	.08
Liver pathology					
Biliary atresia	118 (64.5)	95 (67.4)	23 (54.8)	1.00 (reference)	
Other	65 (35.5)	46 (32.6)	19 (45.24)	7.71 (0.85–3.44)	.14
Cirrhosis	157 (85.8)	122 (86.5)	35 (83.3)	0.78 (0.30–2.00)	.60
ASAT (U/L)	155 [85–231]	171 [95–221]	125 [82–228]	1.00 (0.99–1.00)	.21
ALAT (U/L)	89 [49–131]	94 [52–134]	80 [45–125]	1.00 (0.99–1.00)	.42
Bilirubin (mg/dL)	15.1 [2.8–20.3]	15.1 [2.9–21.1]	14.3 [2.7–23.1]	1.00 (0.97–1.03)	.79
INR	1.26 [1.09–1.60]	1.26 [1.09–1.61]	1.26 [1.08–1.56]	0.83 (0.45–1.53)	.56
Urea (mg/dL)	16 [13–22]	16 [12–21]	16 [13–23]	1.00 (0.96–1.04)	.97
Creatinine (mg/dL)	0.25 [0.19–0.33]	0.24 [0.17–0.32]	0.29 [0.23–0.36]	22.02 (1.25–399.21)	**.03**
Albumin (g/L)	32 [29–38]	32 [28–37]	35 [32–39]	1.04 (0.99–1.09)	.08
NLR	1.0 [0.6–1.5]	0.9 [0.6–1.4]	1.2 [0.7–2.1]	1.35 (1.04–1.75)	**.03**

Data are presented as median [IQR] or numbers (%). Logistic regression was used to estimate odds ratios with 95% confidence intervals for IPGF

*Abbreviations*: *ASAT* Aspartate aminotransferase, *ALAT* Alanine aminotransferase, *NLR* Neutrophil/lymphocyte ratio

**Table 4:** Intraoperative characteristics of the whole cohort and unadjusted odds ratios for initial poor graft function

	**Whole cohort (*n* = 183)**	**No IPGF (*n* = 141)**	**IPGF (*n* = 42)**	**OR for IPGF (95%CI)**	***P* value**
**Donor**					
Hypnotic					
Sevoflurane	69 (37.7)	58 (41.1)	11 (26.2)	1.00 (reference)	
Propofol	85 (46.4)	66 (46.8)	19 (45.2)	1.69 (0.67–3.45)	.32
Propofol + sevoflurane	29 (15.8)	17 (12.1)	12 (28.6)	3.72 (1.40–9.92)	**.0008**
NSAIDs	143 (78.1)	111 (78.7)	32 (76.2)	0.86 (0.38–1.96)	.73
Sufentanil	71 (38.8)	60 (42.6)	11 (26.2)	0.48 (0.22–1.03)	.06
Clonidine	136 (74.3)	104 (79.8)	32 (76.2)	1.14 (0.51–2.54)	.75
Ketamine	178 (97.3)	136 (96.5)	42 (100.0)	NA^a^	NA^a^
Epidural	152 (83.1)	117 (83.0)	35 (83.3)	1.03 (0.41–2.58)	.96
Noradrenalin use	50 (27.3)	37 (26.2)	13 (31.0)	1.26 (0.59–2.68)	.55
**Receiver**					
Procedure duration (min)	648 [589–699]	642 [583–696]	675 [638–725]	1.45 (1.09–1.91)	**.009**
Ischemia duration (min)	143 [123–170]	142 [120–168]	147 [125–180]	1.01 (0.99–1.02)	.16
Vasopressor use	158 (86.3)	120 (85.1)	38 (90.5)	1.66 (0.54–5.15)	.38
Lactate peak (mmol/L)	5.1 [4–6.8]	5.3 [4.1–7.0]	5.1 [3.9–6.2]	0.98 (0.85–1.14)	.84
Reperfusion syndrome	31 (16.9)	24 (17.0)	7 (16.7)	0.98 (0.39–2.45)	.96
Graft weight/body weight (%)	2.7 [1.9–3.5]	2.8 [2.0–3.7]	2.1 [1.7–3.2]	0.71 (0.51–0.99)	**.03**

Data are presented as median [IQR] or numbers (%). Logistic regression was used to estimate odds ratios with 95% confidence intervals for IPGF

*Abbreviation*: *NSAIDs* Non-steroidal anti-inflammatory drugs

^a^Unstable estimate as there were no pairs where the donor received ketamine and the recipient developed IPGF

### Factors associated with initial poor graft function in multivariate analysis

The factors independently associated with the occurrence of IPGF after backward variable selection and multivariate logistic regression were the duration of the transplantation surgery (*p* = 0.009), the anesthetic agent used for maintenance in the donor (*p* = 0.004), the preoperative NLR (*p* = 0.02) and albumin (*p* = 0.05) in the recipient, and the age of the donor (*p* = 0.03) (Table 5). More specifically, recipients in the propofol-sevoflurane group were significantly more likely to experience IPGF than recipients in the sevoflurane group (OR 4.93, CI 1.65–14.72, *p* = 0.004). While recipients in the propofol group seemed more likely to develop IPGF than those in the sevoflurane group, this difference did not reach statistical significance (OR 2.13, CI 0.84–5.40, *p* = 0.11).

**Table 5:** Multivariable-adjusted odds ratios for initial poor graft function after variable selection

	**OR (95% CI)**	***P* value**
**Donor**		
Age (years)	1.07 (1.01–1.15)	**.03**
Hypnotic		
Sevoflurane	1.00 (reference)	
Propofol	2.13 (0.84–5.40)	.11
Propofol + sevoflurane	4.92 (1.65–14.72)	**.004**
**Receiver**		
NLR	1.44 (1.07–1.95)	**.02**
Procedure duration (hours)	1.55 (1.12–2.16)	**.009**
Albumin (g/L)	1.06 (1.00–1.13)	.**05**

*Abbreviation*: *NLR* Neutrophil/lymphocyte ratio

Multiple logistic regression was used to estimate odds ratios with 95% confidence intervals for IPGF, after variable selection with a backward stepwise method (entry criteria: *p* < .2, exit criteria: *p* < .1)

## Discussion

Our study’s main finding is that when the maintenance of anesthesia was performed by sevoflurane in living liver donors from the induction to the harvesting of the graft, significantly fewer children developed IPGF.

So far, only one study has evaluated the preconditioning effect of sevoflurane on EAD in liver transplantation (Minou et al. 2012). In a randomized controlled trial, sevoflurane preconditioning was administered to brain-dead donors and showed a significant decrease in EAD compared to the control group, an effect we also found in our study. However, the authors noticed that this positive effect was only apparent in the case of steatosis. In our cohort, sevoflurane appears superior in grafts originating from living donors, a population particularly selected for its good health and absence of liver steatosis.

Two studies compared the effect on early graft function of sevoflurane and propofol administered as postconditioning (Beck-Schimmer et al. 2015; Gajate Martin et al. 2016). In a retrospective study comparing sevoflurane to propofol anesthesia in recipients, Gajate Martín et al. found no difference regarding EAD (Gajate Martin et al. 2016). However, it is interesting that they used a more straightforward definition of EAD, which included only transaminase levels. In contrast, the composite definition typically used by the other studies investigating this topic includes INR and bilirubin levels (Olthoff et al. 2010; Beck-Schimmer et al. 2015; Minou et al. 2012; Nguyen et al. 2019). This lack of difference in transaminase level between both groups is in line with the results of a prospective randomized controlled trial (RCT) carried out a year earlier by Beck-Schimmer et al. (2015). However, they mentioned that the incidence of EAD, using the composite definition, was lower in the sevoflurane group, but without reaching statistical significance (Beck-Schimmer et al. 2015). Besides the small sample size, the authors highlighted two other possible explanations for their negative findings. Their sevoflurane regimen may have had no effect because it had only been applied as a postconditioning strategy. Indeed, due to logistic issues, the transplanted cadaveric grafts were not exposed to volatile anesthetics before cross-clamping. They also suggested that organ injuries in transplantation are caused by various factors, including donor characteristics that may constitute confounding factors. In our study, donors were a homogeneous group of young, healthy people instead of a brain-dead donor population. This may have led to better standardization, enabling the demonstration of a potential beneficial effect of sevoflurane on IRI. Of note, all the grafts benefited from pharmacologic postconditioning since all children received sevoflurane throughout the surgical procedure.

The effects of sevoflurane preconditioning have also been investigated in non-transplant liver surgery, with conflicting results. In an RCT, Beck-Schimmer et al. demonstrated that sevoflurane anesthesia significantly limited the postoperative increase of transaminase levels after hepatectomy with inflow occlusion (Beck-Schimmer et al. 2008). This benefit was more pronounced in patients with steatosis. On the contrary, in another RCT investigating pharmacological conditioning during hepatectomy with inflow occlusion, Song et al. did not show any difference in transaminase levels and clinical outcomes between the propofol and sevoflurane groups (Song et al. 2010). A possible explanation for these different findings may lie in the different durations of ischemia. Indeed, the average duration of Pringle’s maneuver was around 20 min in Song’s study versus 35 min in Beck-Schimmer’s trial. However, it must be emphasized that the ischemia-reperfusion insult observed during a short Pringle’s maneuver in liver resection surgery is less important than that observed in transplantation surgery. Hence, results from liver resection surgery must be extrapolated with caution. The longer duration of ischemic stress observed in transplantation surgery could have a greater potential to reveal the beneficial effects of an IRI prevention strategy.

Interestingly, in our study, the association of propofol and sevoflurane in the donor did not demonstrate any advantage in preserving the graft’s function compared to sevoflurane alone, suggesting, among other hypotheses, that the effect of sevoflurane might be dose-dependent. Several preclinical studies have investigated this question and the optimal timing of sevoflurane administration to protect graft function. In an animal model, Zhou et al. demonstrated a protective effect of sevoflurane preconditioning against hepatic IRI, but no significant dose-response relationship was found (Zhou et al. 2013). The benefit appeared to be the same for 1, 1.5, and 2 MAC. The authors concluded that a dose-response relationship might exist at lower concentrations, evoking a threshold effect that had previously been demonstrated by Obal et al*.* in a rat heart model, highlighting that preconditioning with sevoflurane at 1.0 MAC offered better protection than 0.75 MAC but that there was no additional benefit to increase the dose beyond 1.0 MAC (Obal et al. 2001). In addition to the dose, the duration of exposure to sevoflurane could also play an important role, and there may be an additive effect of pre- and postconditioning (De Hert et al. 2004; Zitta et al. 2010). Nevertheless, no clear consensus can currently be reached on the optimal timing of sevoflurane administration, and the dose-dependency of the pharmacological preconditioning effect still needs to be determined.

Another interesting finding of our study is that preoperative NLR in the recipient is also an independent predictor of IPGF. For several years, NLR has been known as an indicator of systemic inflammatory status, reflecting the balance between innate and adaptive immune function. The association between preoperative NLR and EAD was already known and described in adult liver transplantation after both cadaveric and living donation (Kwon et al. 2019; Nylec et al. 2020). It is explained by the crucial role of the inflammatory response and the preoperative immune status of the recipient in the development of EAD (Oweira et al. 2016). This is the first time these results have been confirmed in a pediatric cohort.

Another factor associated with IPGF in our study is donor age, a result already reported in several studies. In a retrospective study of 300 deceased donors, Olthoff et al. found an adjusted OR of 3.12 for the development of EAD in donors aged > 45 years (Olthoff et al. 2010). These results are consistent with earlier findings identifying donor age > 49 as an independent risk factor for EAD and primary non-function (Ploeg et al. 1993). It must be noted that deceased donors are often older than patients selected for a living donation. Interestingly, our study shows that donor age is significantly associated with IPGF, even in a young population with a median age of 34. These findings align with the results of another retrospective study that found an association between donor age and EAD in the adult-to-adult LDLT setting (Pomposelli et al. 2016).

Finally, we identified the duration of transplant surgery as a predictive factor for IPGF. At the same time, prolonged warm and cold ischemia times are known to induce more severe IRI (Ito et al. 2021). Surgery time as such is rarely mentioned in the literature. Still, in a retrospective study published in 2007, the authors found an association between operating room time and primary graft nonfunction, often leading to retransplantation (Uemura et al. 2007). It can be assumed that the harmful effects of prolonged surgery time are at least partially linked to prolonged ischemia time but also to more complex surgical conditions, as found in patients with PHT or severe coagulopathy.

This study suffers from several limitations inherent to its retrospective nature. First, we had a relatively high percentage (17.8%) of patient pairs with missing data. However, we analyzed the missing values in the dataset according to recently published recommendations. Since missingness was not associated with the outcome or the predictors, a complete case analysis has been shown to provide unbiased estimates (Bartlett et al. 2015; Lee et al. 2021). A second limitation—common to most studies investigating this topic—is the choice of surrogate biological markers instead of a clinical outcome. Indeed, if the release of liver transaminase is a clinical marker for acute graft injury, its clinical significance remains unclear. Third, model overfitting is a common potential problem in risk factor analyses, especially when the sample size is relatively limited. Hence, we acknowledge that our findings should be validated on an external database in order to support their robustness and generalizability. Further clinical studies, especially prospective randomized controlled trials, are needed to confirm the positive effect of sevoflurane preconditioning in liver transplantation. Fourth, the collected data covers a decade during which practices have constantly evolved, both from a surgical and an anesthetic point of view. Finally, in our cohort, there were differences between the sevoflurane, propofol, and propofol-sevoflurane groups both in donor and receiver intraoperative variables. However, none of these variables were associated with the occurrence of IPGF in multivariate logistic regression.

Nevertheless, the strength of this study lies in the fact that it is the first to investigate pharmacological preconditioning with sevoflurane in LDLT and also the first study investigating the impact of sevoflurane preconditioning on graft function in a pediatric population.

## Conclusion

Preconditioning with sevoflurane in living liver donors could be a promising intervention to improve initial graft function in pediatric recipients. Although randomized controlled trials are required to confirm its benefits, sevoflurane can be applied without any significant adverse effects and may provide a tool to protect early graft function following living liver donation.

### Supplementary Information

**Additional file 1: Supplementary Table S1.** Summary of the study variables, including the amount of data available for each variable, and a summary of the characteristics for the enrolled sample and those with complete records. **Supplementary Table S2.** Predictors of being a complete case in the current study. **Supplementary Table S3.** Postoperative evolution of individual biological outcomes according to the anesthetic agent used in the donor.

## Data Availability

The datasets used and analyzed during the current study are available from the corresponding author (Audrey Dieu) at reasonable request.
