# Supplementary material for: Sevoflurane preconditioning in living liver donation is associated with better initial graft function after pediatric transplantation: a retrospective study
Source: Perioper Med (Lond). 2024 Feb 28;13:11. doi: 10.1186/s13741-024-00367-x (PMC10903053; doi:10.1186/s13741-024-00367-x)
Supplement: Supplementary file 1 — Additional file 1: Supplementary Table S1. Summary of the study variables, including the amount of data available for each variable, and a summary of the characteristics for the enrolled sample and those with complete records. Supplementary Table S2. Predictors of being a complete case in the current study. Supplementary Table S3. Postoperative evolution of individual biological outcomes according to the anesthetic agent used in the donor. [file 13741_2024_367_MOESM1_ESM.docx]

# Supplementary Tables

Supplementary Table 1 – Summary of the study variables, including the amount of data available for each variable, and a summary of the characteristics for the enrolled sample and those with complete records.

|  |  | **Available data**  **(n = 222)** | | **Complete cohort  (n = 222)*** | | **Complete cases (n = 183)** | |
| --- | --- | --- | --- | --- | --- | --- | --- |
| **Preoperative** |  |  | |  | |  | |
| **Donor** |  |  | |  | |  | |
| Age (years) | Median [IQR] | 222 | (100) | 34.0 | [29.1–37.4] | 34.0 | [28.8–37.1] |
| Sex | Female | 222 | (100) | 101 | (45.5) | 85 | (46.4) |
|  | Male |  |  | 121 | (54.5) | 98 | (53.6) |
| **Recipient** |  |  |  |  |  |  |  |
| Age (years) | Median [IQR] | 222 | (100) | 1.6 | [0.8–3.3] | 1.6 | [0.9–2.8] |
| Sex | Female | 222 | (100) | 125 | (56.3) | 103 | (56.3) |
|  | Male |  |  | 98 | (43.7) | 80 | (43.7) |
| Weight (kg) | Sex | 222 | (100) | 9.5 | [7.5–14.0] | 9.6 | [7.6–14.0] |
| Cirrhosis | Yes | 222 | (100) | 188 | (84.7) | 157 | (85.8) |
|  | No |  |  | 34 | (15.3) | 26 | (14.2) |
| ASAT (U/L) | Median [IQR] | 222 | (100) | 153 | [89–219] | 155 | [85–231] |
| ALAT (U/L) | Median [IQR] | 222 | (100) | 85 | [49–130] | 89 | [49–131] |
| Bilirubin (mg/dL) | Median [IQR] | 221 | (99.5) | 15.0 | [2.7–21.4] | 15.1 | [2.8–20.3] |
| INR | Median [IQR] | 222 | (100) | 1.3 | [1.1–1.6] | 1.26 | [1.09–1.60] |
| Urea (mg/dL) | Median [IQR] | 222 | (100) | 16.0 | [12.0–22.2] | 16 | [13–22] |
| Creatinin (mg/dL) | Median [IQR] | 222 | (100) | 0.3 | [0.2–0.3 | 0.25 | [0.19–0.33] |
| Albumin (g/L) | Median [IQR] | 218 | (98.2) | 33.0 | [29.0–38.0] | 32 | [29–38] |
| NLR | Median [IQR] | 221 | (99.5) | 1.0 | [0.6–1.6] | 1.0 | [0.6–1.5] |
| **Intraoperative** |  |  | |  | |  | |
| **Donor** |  |  | |  | |  | |
| Anesthetic | Sevoflurane | 220 | (99) | 79 | (35.9) | 29 | (15.8) |
|  | Propofol | 220 | (99) | 104 | (47.3) | 85 | (46.4) |
|  | Propofol-sevoflurane | 220 | (99) | 37 | (16.8) | 69 | (37.7) |
| NSAIDs | Yes | 220 | (99) | 175 | (79.6) | 143 | (78.1) |
|  | No |  |  | 45 | (20.4) | 40 | (22.9) |
| Sufentanil | Yes | 220 | (99) | 83 | (37.7) | 71 | (38.8) |
|  | No |  |  | 137 | (62.3) | 112 | (61.2) |
| Clonidine | Yes | 220 | (99) | 168 | (76.4) | 136 | (74.3) |
|  | No |  |  | 52 | (23.6) | 47 | (25.7) |
| Ketamine | Yes | 220 | (99) | 215 | (97.7) | 178 | (97.3) |
|  | No |  |  | 5 | (2.3) | 5 | (2.7) |
| Epidural | Yes | 220 | (99) | 181 | (82.3) | 152 | (83.1) |
|  | No |  |  | 39 | (17.7) | 31 | (17.9) |
| Noradrenalin | Yes | 220 | (99) | 53 | (24.1) | 50 | (27.3) |
|  | No |  |  | 167 | (75.9) | 153 | (72.7) |
| **Recipient** |  |  |  |  |  |  |  |
| Procedure duration (min) | Median [IQR] | 214 | (96) | 648.0 | [585–698] | 648 | [589–699] |
| Ischemia duration (min) | Median [IQR] | 218 | (98) | 145 | [123–171] | 143 | [123–170] |
| Vasopressor use | Yes | 213 | (96) | 183 | (85.9) | 158 | (86.3) |
|  | No |  |  | 30 | (14.1) | 25 | (13.7) |
| Lactate peak (mmol/L) | Median [IQR] | 219 | (99) | 5.2 | [4–6.8] | 5.1 | [4–6.8] |
| Reperfusion syndrome | Yes | 213 | (96) | 38 | (17.8) | 31 | (16.9) |
|  | No |  |  | 175 | (82.2) | 152 | (83.1) |
| Graft weight/body weight (%) | Median [IQR] | 199 | (90) | 2.7 | [1.9–3.5] | 2.7 | [1.9–3.5] |
| **Outcome** |  |  |  |  |  |  |  |
| Initial poor graft function | Yes | 222 | (100) | 53 | (76.1) | 42 | (22.9) |
|  | No |  |  | 169 | (23,9) | 141 | (77.1) |

ASAT: aspartate amino transferase, ALAT: alanine amino transferase, NLR: neutrophil/lymphocyte ratio, INR: International Normalized Ratio, NSAIDs: non-steroidal anti-inflammatory drugs. Data are presented as median [IQR] or numbers. *Denominators vary because some variables have different completion rates.

Supplementary Table 2 – Predictors of being a complete case in the current study.

|  |  | **OR (95%CI)** | **AUC** |
| --- | --- | --- | --- |
| **Preoperative** |  |  |  |
| **Donor** |  |  |  |
| Age (years) | Per unit change | 0.98 (0.93–1.03) | 0.55 |
| Sex | Female | 1.00 (reference) | 0.53 |
|  | Male | 0.80 (0.39–1.62) |  |
| **Recipient** |  |  |  |
| Age (years) | Per unit change | 1.001 (0.89–1.14) | 0.52 |
| Sex | Female | 1.00 (reference) | 0.50 |
|  | Male | 1.01 (0.50–2.02) |  |
| Weight (kg) | Per unit change | 1.02 (0.96–1.08) | 0.54 |
| Cirrhosis | Yes | 1.00 (reference) | 0.53 |
|  | No | 0.64 (0.27–1.55) |  |
| ASAT (U/L) | Per unit change | 1.00 (0.99–1.00) | 0.55 |
| ALAT (U/L) | Per unit change | 1.00 (0.99–1.01) | 0.53 |
| Bilirubin (mg/dL) | Per unit change | 0.99 (0.96–1.02) | 0.51 |
| INR | Per unit change | 0.94 (0.55–1.60) | 0.50 |
| Urea (mg/dL) | Per unit change | 0.99 (0.95–1.03) | 0.52 |
| Creatinin (mg/dL) | Per unit change | 0.44 (0.12–1.62) | 0.47 |
| Albumin (g/L) | Per unit change | 0.98 (0.93–1.03) | 0.54 |
| NLR | Per unit change | 0.94 (0.87–1.02) | 0.56 |
| **Intraoperative** |  |  |  |
| **Donor** |  |  |  |
| Anesthetic | Sevoflurane | 1.00 (reference) | 0.56 |
|  | Propofol | 0.64 (0.28–1.49) |  |
|  | Propofol-sevoflurane | 0.52 (0.19–1.46) |  |
| NSAIDs | Yes | 1.00 (reference) | 0.54 |
|  | No | 1.79 (0.65–4.89) |  |
| Sufentanil | Yes | 1.00 (reference) | 0.53 |
|  | No | 0.76 (0.36–1.60) |  |
| Clonidine | Yes | 1.00 (reference) | 0.56 |
|  | No | 2.21 (0.81–6.01) |  |
| Ketamine | Yes | 1.00 (reference) | NA* |
|  | No | NA* |  |
| Epidural | Yes | 1.00 (reference) | 0.52 |
|  | No | 0.74 (0.31–1.77) |  |
| Noradrenalin | Yes | 1.00 (reference) | 0.59 |
|  | No | 0.24 (0.06-0.79) |  |
| **Recipient** |  |  |  |
| Procedure duration (min) | Per unit change | 1.00 (0.99–1.01) | 0.58 |
| Ischemia duration (min) | Per unit change | 0.99 (0.99–1.00) | 0.54 |
| Vasopressor use | Yes | 1.00 (reference) | 0.52 |
|  | No | 0.79 (0.28–2.26) |  |
| Lactate peak (mmol/L) | Per unit change | 1.06 (0.89–1.25) | 0.52 |
| Reperfusion syndrome | Yes | 1.00 (reference) | 0.53 |
|  | No | 1.49 (0.59–3.78) |  |
| Graft weight/body weight (%) | Per unit change | 0.79 (0.53–1.19) | 0.55 |
| **Outcome** |  |  |  |
| Initial poor graft function | Yes | 1.00 (reference) | 0.53 |
|  | No | 1.32 (0.61–2.87) |  |

Abbreviations: ASAT: aspartate aminotransferase, ALAT: alanine aminotransferase, INR: International Normalized Ratio, NLR: neutrophil/lymphocyte ratio. NSAIDs: non-steroidal anti-inflammatory drugs. Logistic regression was used to estimate odds ratios with 95% confidence intervals for being a complete case. *Unstable estimate.

Supplementary Table 3 – Postoperative evolution of individual biological outcomes according to the anesthetic agent used in the donor.

|  | **Sevoflurane  (n = 69)** | | **Propofol and sevoflurane  (n = 29)** | | **Propofol (n = 85)** | |
| --- | --- | --- | --- | --- | --- | --- |
| **Postoperative day 1** | | | | | | |
| ASAT (U/L) | 322 | [240–509] | 489 | [287–713] | 435 | [302–612] |
| ALAT (U/L) | 322 | [221–473] | 445 | [235–858] | 382 | [234–646] |
| Bilirubin (mg/dL) | 6.6 | [3.4–9.2] | 7.2 | [4.2–9.4] | 6.5 | [3.9–9.2] |
| INR | 1.88 | [1.56–2.33] | 2.13 | [1.88–2.52] | 1.84 | [1.59–2.17] |
| Urea (mg/dL) | 20 | [14–32] | 26 | [21–34] | 25 | [21–34] |
| Creatinin (mg/dL) | 0.17 | [0.17–0.21] | 0.21 | [0.14–0.26] | 0.19 | [0.14–0.30] |
| NLR | 2.6 | [1.8–4.0] | 3.3 | [1.8–5.8] | 3.6 | [2.3–5.0] |
| **Postoperative day 2** | | | | | | |
| ASAT (U/L) | 202 | [159–296] | 258 | [196–506] | 250 | [186–342] |
| ALAT (U/L) | 273 | [198–398] | 325 | [189–741] | 328 | [238–466] |
| Bilirubin (mg/dL) | 5.2 | [2.9–7.1] | 4.7 | [3.1–7.9] | 5.2 | [3.2–7.4] |
| INR | 2.19 | [1.79–2.78] | 2.33 | [1.98–2.97] | 1.85 | [1.55–2.28] |
| Urea (mg/dL) | 23 | [18–35] | 41 | [24–48] | 31 | [19–43] |
| Creatinin (mg/dL) | 0.17 | [0.17–0.20] | 0.22 | [0.12–0.31] | 0.17 | [0.10–0.30] |
| NLR | 2.2 | [1.5–3.2] | 1.9 | [1.4–4.0] | 2.3 | [1.6–3.4] |
| **Postoperative day 3** | | | | | | |
| ASAT (U/L) | 107 | [82–144] | 135 | [110–2010] | 131 | [99–186] |
| ALAT (U/L) | 203 | [148–292] | 286 | [133–561] | 265 | [175–333] |
| Bilirubin (mg/dL) | 4.9 | [3.0–7.3] | 5.8 | [3.0–9.7] | 4.5 | [2.6–7.8] |
| INR | 1.47 | [1.29–1.83] | 1.67 | [1.48–2.04] | 1.36 | [1.18–1.59] |
| Urea (mg/dL) | 25 | [18–43] | 38 | [23–53] | 27 | [16–43] |
| Creatinin (mg/dL) | 0.17 | [0.17–0.24] | 0.19 | [0.14–0.37] | 0.18 | [0.10–0.25] |
| NLR | 1.7 | [1.0–3.0] | 1.5 | [1.1–2.9] | 1.7 | [1.0–2.5] |
| **Postoperative day 4** | | | | | | |
| ASAT (U/L) | 66 | [45–101] | 82 | [73–146] | 80 | [59–112] |
| ALAT (U/L) | 163 | [109–215] | 205 | [113–467] | 188 | [140–257] |
| Bilirubin (mg/dL) | 4.3 | [2.8–7.6] | 5.1 | [2.7–8.1] | 4.2 | [2.1–7.0] |
| INR | 1.37 | [1.15–1.57] | 1.50 | [1.31–1.83] | 1.24 | [1.15–1.51] |
| Urea (mg/dL) | 20 | [12–41] | 31 | [13–42] | 18 | [11–34] |
| Creatinin (mg/dL) | 0.17 | [0.17–0.20] | 0.18 | [0.11–0.30] | 0.14 | [0.09–0.24] |
| NLR | 1.5 | [0.8–2.5] | 1.4 | [0.9–2.5] | 1.2 | [0.9–2.1] |
| **Postoperative day 5** | | | | | | |
| ASAT (U/L) | 63 | [37–81] | 69 | [46–94] | 68 | [45–100] |
| ALAT (U/L) | 122 | [86–169] | 155 | [77–314] | 150 | [108–216] |
| Bilirubin (mg/dL) | 3.6 | [2.3–6.1] | 4.7 | [2.2–8.8] | 3.8 | [2.1–6.8] |
| INR | 1.35 | [1.17–1.58] | 1.50 | [1.29–1.80] | 1.25 | [1.13–1.45] |
| Urea (mg/dL) | 13 | [8–24] | 23 | [13–40] | 12 | [8–22] |
| Creatinin (mg/dL) | 0.17 | [0.17–0.20] | 0.19 | [0.15–0.35] | 0.14 | [0.09–0.19] |
| NLR | 1.4 | [0.9–2.2] | 1.5 | [0.8–2.2] | 1.3 | [0.9–2.2] |
| **Postoperative day 6** | | | | | | |
| ASAT (U/L) | 61 | [32–85] | 51 | [41–91] | 64 | [37–88] |
| ALAT (U/L) | 106 | [68–142] | 126 | [61–212] | 136 | [87–178] |
| Bilirubin (mg/dL) | 3.0 | [2.1–6.7] | 4.5 | [1.8–7.5] | 3.3 | [1.8–5.9] |
| INR | 1.40 | [1.23–1.59] | 1.40 | [1.25–1.68] | 1.27 | [1.13–1.47] |
| Urea (mg/dL) | 12 | [8–20] | 17 | [9–30] | 10 | [7–17] |
| Creatinin (mg/dL) | 0.17 | [0.14–0.21] | 0.17 | [0.14–0.27] | 0.15 | [0.09–0.21] |
| NLR | 1.4 | [0.9–2.4] | 1.6 | [0.8–2.4] | 1.5 | [0.9–2.5] |
| **Postoperative day 7** | | | | | | |
| ASAT (U/L) | 46 | [31–69] | 44 | [33–60] | 51 | [35–74] |
| ALAT (U/L) | 76 | [55–120] | 83 | [54–169] | 109 | [64–146] |
| Bilirubin (mg/dL) | 2.6 | [1.7–5.7] | 3.0 | [1.4–7.0] | 2.7 | [1.5–4.8] |
| INR | 1.37 | [1.23–1.51] | 1.34 | [1.20–1.62] | 1.22 | [1.13–1.45] |
| Urea (mg/dL) | 14 | [7–23] | 13 | [7–27] | 11 | [7–19] |
| Creatinin (mg/dL) | 0.17 | [0.16–0.21] | 0.17 | [0.11–0.24] | 0.16 | [0.10–0.21] |
| NLR | 1.4 | [1.0–2.4] | 1.6 | [0.9–2.0] | 1.4 | [1.0–2.8] |
| **Postoperative day 14** | |  |  |  |  |  |
| ASAT (U/L) | 39 | [32–57] | 38 | [26–58] | 42.5 | [31–61] |
| ALAT (U/L) | 33 | [22–67] | 38 | [24.5–62] | 40.5 | [27–86] |
| Bilirubin (mg/dL) | 1.0 | [0.6–1.7] | 0.8 | [0.5–1.7] | 1.2 | [0.7–1.8] |
| INR | 1.19 | [1.05–1.39] | 1.19 | [1.09–1.34] | 1.14 | [0.99–1.30] |
| Urea (mg/dL) | 23 | [16–31] | 23 | [15–39] | 21 | [14–29] |
| Creatinin (mg/dL) | 0.17 | [0.17–0.19] | 0.19 | [0.11–0.25] | 0.16 | [0.10–0.22] |
| NLR | 1.2 | [0.7–2.1] | 1.6 | [0.9–3.7] | 1.1 | [0.6–2.1] |

Abbreviations: ASAT: aspartate aminotransferase, ALAT: alanine aminotransferase, INR: International Normalized Ratio, NLR: neutrophil/lymphocyte ratio. Data are presented as median [IQR].
